# Dental-amalgam exposure and the risk and progression of multiple sclerosis

**DOI:** 10.1093/ije/dyag059

**Published:** 2026-04-24

**Authors:** Jie Guo, Tomas Olsson, Lars Alfredsson, Anna Karin Hedström

**Affiliations:** Department of Nutrition and Health, China Agricultural University, Beijing, China; Department of Clinical Neuroscience, Karolinska Institutet, Stockholm, Sweden; Institute of Environmental Medicine, Karolinska Institutet, Stockholm, Sweden; Centre for Occupational and Environmental Medicine, Region Stockholm, Stockholm, Sweden; Department of Clinical Neuroscience, Karolinska Institutet, Stockholm, Sweden

**Keywords:** amalgam, mercury, multiple sclerosis risk, multiple sclerosis disability progression

## Abstract

**Background:**

Concerns about the neurotoxic potential of dental amalgam in multiple sclerosis (MS) persists but its impact on disease risk and progression remains unclear.

**Methods:**

We used data from a Swedish population-based case–control study (2386 cases, 4849 controls) to investigate the association between dental amalgam exposure and MS risk. Odds ratios (ORs) with 95% confidence intervals (CIs) were estimated by using logistic regression and interaction with smoking was evaluated. Relapsing-onset MS cases born between 1965 and 1985 (*n* = 1191) were followed longitudinally by using clinical data from the Swedish MS registry. The time to confirmed disability worsening (CDW) and Expanded Disability Status Scale (EDSS) 3 and 4 were assessed by using Cox regression; EDSS trajectories were analysed by using linear mixed-effects models.

**Results:**

Amalgam exposure was associated with increased MS risk in a dose-dependent manner (OR for trend 1.14, 95% CI 1.08–1.20). A significant interaction with smoking was observed (attributable proportion due to interaction 0.25, 95% CI 0.10–0.40). In the longitudinal cohort, those with at least six fillings had increased risk of CDW [hazard ratio (HR) 1.35, 95% CI 1.07–1.71] and progression to EDSS 3 (HR 1.48, 95% CI 1.07–2.05) and EDSS 4 (1.68, 95% CI 1.01–2.83). Associations were stronger among current smokers, those diagnosed after age 40 years, and individuals on low-efficacy therapies. The EDSS trajectories also showed faster progression in the high-exposure group (*P *= .044).

**Conclusion:**

Dental amalgam exposure may be associated with both increased risk of MS and faster disability progression. Findings support a potential synergistic effect with smoking and raise the hypothesis that mercury may contribute to MS-related neurodegeneration.

Key Messages

- We investigated whether exposure to dental amalgam is associated with an increased risk of developing multiple sclerosis (MS) and with faster disability progression.
- Our findings show a dose-dependent increase in MS risk and more rapid disability worsening among those with higher amalgam exposure, particularly smokers.
- These results indicate a potential role of mercury exposure in MS pathogenesis and progression, suggesting that reducing amalgam exposure might improve long-term outcomes.

## Background

Multiple sclerosis (MS) is a chronic, immune-mediated disease of the central nervous system, characterized by inflammation, demyelination, and neurodegeneration. Its etiology involves a combination of genetic predisposition and environmental exposures [1]. Beyond established risk factors such as Epstein–Barr virus infection [2] and smoking [3], other potential exposures have been considered, including mercury released from dental amalgam.

Dental amalgam, widely used for decades as a restorative material, contains ∼50% elemental mercury and emits low levels of mercury vapor that can be inhaled and absorbed [4, 5]. Given the neurotoxicity and tissue accumulation of mercury, concerns about possible long-term effects on the central nervous system have persisted [6, 7].

A possible link between dental amalgam and MS was suggested in the 1960s but subsequent studies have yielded inconsistent results. Four studies have reported elevated MS risk with amalgam exposure [8–11], while two found no evidence of an association [12, 13]. Most previous studies have been limited by small samples and residual confounding. One small cross-sectional study (*n* = 33) also reported higher Expanded Disability Status Scale (EDSS) scores in exposed individuals [14].

We used a population-based, incident case–control study to examine the association between dental amalgam exposure and MS risk, and a longitudinal analysis among cases to assess whether amalgam exposure is associated with disability progression over time.

## Method

### Design and study population

The Epidemiological Investigation of Multiple Sclerosis [17] is a population-based case–control study targeting individuals aged 16–70 years residing in Sweden. Incident MS cases were recruited by treating neurologists between April 2005 and December 2014 at 40 neurology clinics nationwide, including all university hospitals. Cases were invited to participate at the time of diagnosis. Eligibility criteria for cases were age 16–70 years, residence in Sweden, and a neurologist-confirmed diagnosis of MS according to the McDonald criteria [15, 16]. For each case, two controls were randomly selected from the national population register by using incidence density sampling, frequency-matched to the case by age in 5-year strata, sex, and residential area.

Participants completed version 1 of an extensive questionnaire at the time of recruitment covering lifestyle and environmental factors, including questions on dental amalgam exposure. Participants who returned questionnaires with missing or ambiguous responses were contacted by study staff to clarify and complete the items. Completed questionnaires were obtained from 2424 cases (91%) and 4899 controls (69%). Participants who were unsure whether they had amalgam fillings (8 cases and 10 controls) or who reported mercury exposure through occupation or education (30 cases and 40 controls) were excluded. The sample for the case–control analysis thus comprised 2386 cases and 4849 controls.

For the longitudinal analysis of disability progression, we included a subset of 1191 cases with relapsing-onset MS who were born between 1965 and 1985. This restriction was applied to reduce the risk of bias due to substantial differences in both amalgam exposure and treatment availability across the birth cohorts (Supplementary Table 1). Amalgam use was phased out in Sweden starting in the 1990s, resulting in very few exposed individuals among younger participants. At the same time, younger patients were more likely to receive high-efficacy disease-modifying treatment (DMT). Conversely, among older participants, nearly all had amalgam fillings and treatment options were limited or unavailable. Restriction of the analysis to those born between 1965 and 1985 allowed greater comparability in both exposure and treatment contexts.

The study was approved by the Regional Ethical Review Board at Karolinska Institute (reference numbers 2004–252/1–4 and 2013/1691–32) and was conducted in accordance with the ethical standards of the 1964 Declaration of Helsinki and its later amendments. All subjects in the study provided written informed consent before participation.

### Dental amalgam exposure

Participants were asked whether they had ever had any dental amalgam fillings. Those who had were asked to report the number of fillings, choosing from the following categories: 0, 1–5, 6–10, 11–15, or >15. In the case–control study of MS risk, amalgam exposure was analysed by using all five categories. In the longitudinal analysis of disability progression, exposure was grouped into three categories to ensure statistical power and interpretability: zero, one to five, or six or more fillings.

### Statistical analysis

#### Risk of MS (case–control study)

Categorical variables were summarized by using frequencies and percentages, and continuous variables by using means and standard deviations (SDs). Unconditional logistic regression was used to estimate odds ratios (ORs) with 95% confidence intervals (CIs) for the association between dental amalgam exposure and MS risk, adjusting for the matching factors and pre-specified covariates. This approach is suitable for frequency-matched designs and retains all eligible participants after exclusions. Analyses were stratified by sex, smoking (current smoking or nonsmoking at disease onset), and MS phenotype (relapsing-onset or progressive-onset MS). Interaction between dental amalgam exposure and current smoking was assessed based on departure from the additivity of effects by using the attributable proportion due to interaction (AP) with 95% CI. An AP of >0 indicates a synergistic interaction.

Analyses were adjusted for age, sex, and residential area in accordance with the study design, as well as for ancestry, smoking, body mass index at age 20 years, past infectious mononucleosis, and sun-exposure habits. A directed acyclic graph (DAG) illustrating the covariate selection is shown in Supplementary Figure 1, with variable definitions detailed in Methods S1.

#### Disability progression (longitudinal study)

Longitudinal data were obtained from the Swedish MS registry, which prospectively records detailed clinical information [18]. Confirmed disability worsening (CDW) was defined as an increase in the EDSS score of ≥1 point from the first recorded EDSS, sustained over two follow-up visits ≥6 months apart (1.5 points if the baseline EDSS was 0 or 0.5 points if the baseline EDSS was ≥5.5).

The time to 24-week CDW and to EDSS 3 and 4 milestones was analysed by using multivariable Cox proportional hazard regression. The follow-up time extended from the baseline (first recorded EDSS) until the outcome of either dropout, death, or the end of follow-up (6 April 2022). The proportional hazard assumption was verified by using Schoenfeld residuals. Trends were evaluated by modeling amalgam exposure as an ordinal variable. Analyses were stratified by age at diagnosis (<40 or ≥40 years), baseline smoking (current smoking or nonsmoking), and treatment regimen (exclusive use of low-efficacy therapies or any high-efficacy therapy during follow-up).

To assess the EDSS trajectories, we used linear mixed-effect models, including fixed effects for amalgam exposure, disease duration since baseline (years), and their interaction, with random effects and slopes for individuals. Predicted mean EDSS scores with 95% CIs were derived at 5, 10, and 15 years by amalgam category.

All longitudinal analyses were adjusted for age at baseline, sex, disease duration, baseline EDSS score, DMT exposure, past infectious mononucleosis, smoking, body mass index, and sun-exposure habits. DMT exposure was captured as the efficacy class of initial DMT (none, platform, or high-efficacy) and the proportion of follow-up time on DMT. A separate DAG for progression analyses is shown in Supplementary Figure 2.

#### Sensitivity analyses

Sensitivity analyses included additional adjustment for educational attainment (proxy for socioeconomic position), alcohol use, fish consumption (dietary proxy), and physical activity. For MS risk, we also ran conditional logistic regression models. To quantify the robustness to unmeasured confounding, we computed E-values for point estimates and the lower 95% CI bounds. To address temporality, risk analyses were repeated and included only participants whose questionnaire was completed within 1 year of symptom onset. Progression analyses were similarly repeated, aligning the amalgam status to the baseline and excluding cases whose questionnaire was completed >1 year after the baseline EDSS. In further sensitivity analyses, DMT exposure was treated as a time-updated variable, splitting person-time at treatment start, end, or switch, and lagging the exposure by 90 days to mitigate reverse causation.

#### Validation of self-reported amalgam exposure

We validated self-reported amalgam exposure by using data from the Swedish National Board of Health and Welfare on the number of intact teeth recorded by dentists. Participants who had a dental visit within 2 years of questionnaire completion were included in the validation sample. Correlations between self-reported number of amalgam fillings and the number of intact teeth were calculated separately for cases and controls. All analyses were conducted by using Statistical Analysis System version 9.2.

## Results

### Risk of MS (case–control study)

Our analyses of dental amalgam exposure and the risk of developing MS included 2386 cases and 4849 controls matched by age, sex, and residential area. The mean age at onset was 34.5 years and the median time from symptom onset to diagnosis was 1 year. Nearly all cases were recruited within 1 year of diagnosis and questionnaires were completed a median of 2 years after symptom onset. The participants’ characteristics are presented in Table 1.

**Table 1:** Characteristics of cases and controls.

	**Total**		Amalgam		No amalgam	
	Cases	Controls	Cases	Controls	Cases	Controls
*N*	2386	4849	1635	3143	751	1706
Age at index (years) [*n* (SD)]	34.5 (10.6)	34.6 (10.7)	38.3 (9.8)	38.6 (9.8)	26.2 (6.7)	27.1 (7.8)
Women [*n* (%)]	1714 (71.8)	3506 (72.3)	1175 (71.9)	2282 (72.6)	539 (71.8)	1224 (71.6)
Nordic [*n* (%)]	1950 (81.7)	3786 (78.1)	1375 (84.1)	2569 (81.7)	575 (76.6)	1217 (71.3)
Post-secondary education [*n* (%)]	1013 (42.5)	2166 (44.7)	677 (41.4)	1436 (45.7)	336 (44.7)	730 (42.8)
Past IM [*n* (%)]	412 (17.3)	481 (9.9)	253 (15.5)	278 (8.9)	159 (21.2)	203 (11.9)
No past IM [*n* (%)]	1737 (72.8)	3907 (80.6)	1227 (75.1)	2565 (81.6)	510 (67.9)	1342 (78.7)
Unsure [*n* (%)]	237 (9.9)	461 (9.5)	155 (9.5)	300 (9.6)	82 (10.9)	161 (9.4)
Never smoker [*n* (%)]	1114 (46.7)	2706 (55.8)	715 (43.7)	1663 (52.9)	399 (53.1)	1043 (61.1)
Current smoker [*n* (%)]	739 (31.0)	1198 (24.7)	518 (31.7)	757 (24.1)	221 (29.4)	441 (25.9)
Past smoker [*n* (%)]	533 (22.3)	945 (19.5)	402 (24.6)	723 (23.0)	131 (17.4)	222 (13.0)
Alcohol user [*n* (%)]	1647 (69.0)	3455 (71.3)	1174 (71.8)	2359 (75.1)	473 (63.0)	1096 (64.2)
Alcohol/week (grams) [*n* (SD)]	44.5 (62.4)	52.6 (75.0)	45.0 (61.9)	53.1 (70.9)	43.4 (63.3)	51.8 (82.1)
Fish-consumption score [*n* (SD)]	3.8 (1.1)	3.9 (1.1)	3.9 (1.1)	4.0 (1.1)	3.7 (1.1)	3.8 (1.1)
Underweight [*n* (%)]	303 (12.7)	694 (14.3)	179 (11.0)	380 (12.1)	124 (16.5)	314 (18.4)
Normal weight [*n* (%)]	1672 (70.1)	3600 (74.2)	1206 (73.8)	2464 (78.4)	466 (62.1)	1136 (66.6)
Overweight [*n* (%)]	303 (12.7)	438 (9.0)	185 (11.3)	236 (7.5)	118 (15.7)	202 (11.8)
Obese [*n* (%)]	108 (4.5)	117 (2.4)	65 (4.0)	63 (2.0)	43 (5.7)	54 (3.2)
Low sun exposure [*n* (%)]	891 (37.3)	1538 (31.7)	640 (39.1)	1051 (33.4)	251 (33.4)	487 (28.6)
Regular physical activity [*n* (SD)]	1239 (51.9)	2606 (53.7)	423 (56.3)	988 (57.9)	816 (49.9)	1618 (51.5)

IM, infectious mononucleosis. Among subjects with amalgam fillings, 32 cases (1.8%) and 49 controls (1.3%) did not know the number of amalgam fillings they had.

Participants who reported occupational or educational mercury exposure prior to disease onset were excluded from the main analysis (30 cases and 40 controls), the majority of whom were dental staff. Among these excluded individuals, the OR of developing MS associated with occupational mercury exposure was 1.70 (95% CI 1.04–2.80) compared with those without such exposure (data not shown).

Overall, dental amalgam exposure was associated with an increased risk of MS (OR 1.31, 95% CI 1.15–1.49) with a significant dose–response trend (OR 1.14, 95% CI 1.08–1.20). An additive interaction between amalgam exposure and smoking was observed. Among current smokers, the trend across the amalgam-exposure levels was stronger (OR for trend 1.22, 95% CI 1.11–1.33) than that among nonsmokers (OR for trend 1.10, 95% CI 1.02–1.16) (Table 2). The AP was 0.25 (95% CI 0.10–0.40), indicating a synergistic effect of combined exposure to amalgam and smoking on MS risk (Table 3).

**Table 2:** Associations between dental amalgam fillings and risk of MS.

Number of fillings	Cases/controls	OR (95% CI)^a^	OR (95% CI)^b^	OR for trend (95% CI)
0	742/1694	1.0 (reference)	1.0 (reference)	
1–5	560/1130	1.18 (1.02–1.35)	1.19 (1.03–1.37)	
6–10	597/1178	1.24 (1.07–1.43)	1.29 (1.11–1.50)	
11–14	283/539	1.31 (1.08–1.58)	1.33 (1.10–1.61)	
≥15	162/241	1.68 (1.33–2.12)	1.75 (1.38–2.23)	1.14 (1.08–1.20)
**Current smokers**				
**Number of fillings**	**Cases/controls**	**OR (95% CI)^a^**	**OR (95% CI)^c^**	**OR for trend (95% CI)**
0	218/436	1.0 (reference)	1.0 (reference)	
1–5	160/273	1.21 (0.93–1.57)	1.23 (0.94–1.61)	
6–10	190/268	1.50 (1.14–1.97)	1.57 (1.19–2.08)	
11–14	93/135	1.50 (1.06–2.11)	1.57 (1.11–2.23)	
≥15	60/69	1.89 (1.25–2.85)	1.97 (1.29–3.01)	1.22 (1.11–1.33)
**Nonsmokers**				
**Number of fillings**	**Cases/controls**	**OR (95% CI)^a^**	**OR (95% CI)^c^**	**OR for trend (95% CI)**
0	523/1258	1.0 (reference)	1.0 (reference)	
1–5	400/856	1.14 (0.98–1.36)	1.16 (0.99–1.37)	
6–10	406/910	1.12 (0.94–1.33)	1.16 (1.01–1.39)	
11–14	190/404	1.20 (0.96–1.51)	1.21 (1.03–1.52)	
≥15	101/172	1.49 (1.12–2.00)	1.56 (1.16–2.09)	1.10 (1.02–1.16)

a Adjusted for age at disease onset, sex, residential area (according to study design), and ancestry.

b Adjusted for age at disease onset, sex, residential area (according to study design), ancestry, smoking status at disease onset, past infectious mononucleosis, body mass index at age 20 years, and sun-exposure habits.

c Adjusted for age, sex, residential area (according to study design), ancestry, past infectious mononucleosis, body mass index at age 20 years, and sun-exposure habits.

**Table 3:** Associations between combinations of current smoking and dental amalgam status and risk of MS.

Current smoking	Amalgam	Cases/controls	OR (95% CI)^a^	OR (95% CI)^b^	AP (95% CI)
–	–	530/1265	1.0 (reference)	1.0 (reference)	
–	+	1117/2386	1.20 (1.03–1.41)	1.20 (1.04–1.39)	
+	–	221/441	1.80 (1.39–2.33)	1.36 (1.11–1.67)	
+	+	518/757	2.31 (1.81–3.00)	2.06 (1.73–2.45)	0.25 (0.10–0.40)

a Adjusted for age at disease onset, sex, residential area (according to study design), and ancestry.

b Adjusted for age at disease onset, residential area (according to study design), ancestry, past infectious mononucleosis, body mass index at age 20 years, and sun-exposure habits.

Sex-stratified analyses showed similar associations in women and men (Supplementary Table 2). In analyses stratified by MS phenotype, the association between amalgam and MS risk was present in both relapsing-onset and progressive-onset MS, although estimates for the latter were less precise due to the small sample size (Supplementary Table 3).

### Disability progression (longitudinal study)

The longitudinal analysis included 1191 individuals with relapsing-onset MS born between 1965 and 1985. The baseline characteristics of the cases are shown in Supplementary Table 4, while the corresponding characteristics of the population controls from the same birth cohort are presented in Supplementary Table 5.

Higher amalgam exposure was associated with an increased risk of disability progression. Compared with participants with no amalgam fillings, those with six or more fillings had a significantly higher risk of CDW [hazard ratio (HR) 1.35, 95% CI 1.07–1.71]. For progression to EDSS 3 and 4, participants with both one to five and more than six fillings had elevated risks compared with the unexposed group (Table 4).

**Table 4:** Associations between dental amalgam exposure and unfavorable outcomes among individuals with relapsing-remitting MS born in 1965–85.

Number of amalgam fillings	*N*	Years (SD)	CDW (%)	HR (95% CI)^a^	HR (95% CI)^b^
0	455	7.3 (4.7)	215 (47)	1.0 (reference)	1.0 (reference)
1–5	410	7.3 (4.9)	224 (55)	1.13 (0.93–1.37)	1.11 (0.90–1.37)
≥6	374	6.7 (4.8)	223 (60)	1.35 (1.11–1.64)	1.35 (1.07–1.71)
**EDSS 3**					
0	375	8.8 (4.6)	106 (28)	1.0 (reference)	1.0 (reference)
1–5	345	8.5 (5.0)	128 (37)	1.34 (1.03–1.76)	1.38 (1.12–1.98)
≥6	295	8.8 (5.0)	116 (39)	1.37 (1.04–1.81)	1.48 (1.07–2.05)
**EDSS 4**					
0	375	10.4 (4.0)	36 (10)	1.0 (reference)	1.0 (reference)
1–5	345	10.4 (4.5)	56 (16)	1.61 (1.04–2.48)	1.66 (1.05–2.64)
≥6	295	10.6 (4.6)	51 (17)	1.60 (1.02–2.51)	1.68 (1.01–2.83)

a Adjusted for baseline age and sex.

b Adjusted for baseline age, sex, disease duration, baseline EDSS, DMT exposure, baseline smoking status, past infectious mononucleosis, body mass index at diagnosis, sun-exposure habits.

The risk of CDW associated with amalgam exposure was observed across the treatment groups but was more pronounced among those who had received only low-efficacy therapies (HR for at least six fillings was 1.63, 95% CI 1.02–2.60) than among those on high-efficacy therapy (HR 1.28, 95% CI 1.00–1.68) (Supplementary Table 6).

When stratified by age at diagnosis, the strongest association was observed among individuals diagnosed after age 40 years (HR for at least six fillings: 2.52, 95% CI 1.08–5.91) while a more modest but significant association was observed for those diagnosed before age 40 years (HR 1.30, 95% CI 1.01–1.67) (Supplementary Table 6). Stratification by smoking status revealed a higher risk of CDW among current smokers with at least six amalgam fillings (HR 1.89, 95% CI 1.09–3.27), while the association was weaker and of borderline significance among nonsmokers (HR 1.28, 95% CI 1.00–1.66) (Supplementary Table 6).

**Figure 1:**
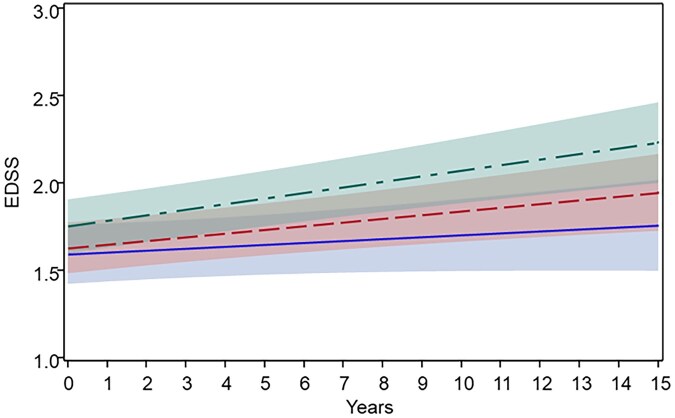
EDSS trajectories post-diagnosis by dental amalgam exposure among individuals with relapsing-onset MS (born in 1965–85). Mean EDSS trajectories (lines) with 95% CIs (shaded) were estimated by using a linear mixed-effects model adjusted for baseline age, sex, disease duration, DMT exposure, baseline smoking status, past infectious mononucleosis, body mass index at diagnosis, and sun-exposure habits. Time 0 denotes the first recorded EDSS score (baseline). Trajectories are shown for individuals with no amalgam (blue), one to five fillings (red), and six or more fillings (green).

The EDSS trajectories over time differed by amalgam-exposure level (Fig. 1). The mean EDSS scores increased in all groups over the 15-year follow-up period but progression was more pronounced among participants with higher amalgam exposure. In the adjusted linear mixed-effects model, individuals with at least six amalgam fillings had a significantly steeper increase in EDSS over time compared with those without amalgam (*β* = 0.02, 95% CI 0.001–0.04, *P* = .044) while the trajectory for the group with one to five fillings did not differ significantly from that for the unexposed group (*β* = 0.01, 95% CI –0.01 to 0.03, *P *= .269) (Supplementary Table 7). The model-predicted mean EDSS at 5, 10, and 15 years showed small absolute differences between the amalgam-exposure groups, consistently with the modest relative hazards (Supplementary Table 8).

### Validation of self-reported amalgam exposure

Dental registry data on number of intact teeth within 2 years of questionnaire completion was available for 68% of the cases and 67% of the controls. Among both groups, a moderate to strong inverse correlation was observed between the number of self-reported amalgam fillings and the number of intact teeth (*r*=−0.6, *P *< .0001), indicating internal consistency and supporting the validity of the self-reported amalgam exposure.

### Sensitivity analyses

Results from our primary models remained similar after further adjustment for educational attainment, fish and alcohol consumption, and physical activity (Supplementary Tables 9 and 10). For MS risk, sensitivity analyses showed consistent ORs and a comparable linear dose–response trend, with an overall OR of 1.31 for any amalgam exposure. The corresponding E-value was 1.95 (lower-bound CI E-value 1.57). For disability progression, sensitivity analyses of CDW showed a HR of 1.35 for high amalgam exposure, with an E-value of 2.04 (lower-bound CI E-value 1.34), indicating that an unmeasured confounder would need risk-ratio associations of approximately two-fold with both amalgam exposure and the outcome, beyond the covariates already included, to fully explain the observed associations. In the sensitivity analyses addressing temporality (restricting the risk analysis to participants with questionnaire completion within 1 year of symptom onset and the longitudinal analysis to cases who completed the questionnaire within 1 year of the baseline EDSS assessment), the estimates were similar to those in the primary analyses (data not shown). The estimates also remained similar when a time-updated (90-day lagged) DMT efficacy class was used, indicating that time-varying treatment did not significantly change the association between amalgam exposure and progression (data not shown). For the case–control analysis of MS risk, conditional logistic regression conditioning on the matching strata yielded effect estimates that were similar to those of the primary unconditional models, with slightly wider CIs due to a loss of participants in the incomplete strata (data not shown).

## Discussion

We observed a dose–response relationship between self-reported dental amalgam exposure and the risk of developing MS, and a synergistic effect between amalgam and smoking, suggesting that combined exposure may amplify the susceptibility to MS. In our longitudinal analysis of individuals with relapsing-onset MS, higher amalgam exposure was also associated with faster disability progression.

While a clear gradient was observed for CDW, the risks for progression to EDSS 3 and 4 were similar in the moderate- and high-exposure groups, suggesting a potential threshold effect rather than a strictly linear dose–response. Associations were most pronounced among individuals receiving low-efficacy treatment and those diagnosed after age 40 years. The strongest effect was observed for progression to EDSS 4, typically reflecting irreversible neurological impairment, suggesting an influence of amalgam on the degenerative component of MS rather than the relapsing–inflammatory phase alone. This interpretation is consistent with evidence implicating oxidative stress, mitochondrial dysfunction, and glial activation [19] in MS neurodegeneration, all of which may be potentiated by chronic low-level mercury exposure [20, 21].

The stronger association among current smokers supports a synergistic effect between amalgam exposure and smoking on MS progression. This aligns with our case–control findings and suggests that smoking may exacerbate mercury-related neurotoxicity and contribute to faster disease worsening [22, 23]. The association between amalgam exposure and disability progression appeared weaker among participants treated with high-efficacy therapies. Whether this reflects reduced vulnerability due to better overall disease control or differences in baseline risk remains uncertain.

The consistency of the findings across the risk and progression analysis, and the parallel results among participants with reported occupational mercury exposure strengthen the evidence for a link between amalgam and MS. While prior studies on amalgam and MS risk have produced mixed results [8–13], many were limited by small sample sizes, limited control for confounding, and variable methodological quality.

Although dental amalgam is no longer used in restorative dentistry in Sweden, most adults still carry existing amalgam fillings. Several European countries have similarly restricted or phased out its use and the US Food and Drug Administration recommends avoiding amalgam in high-risk groups, including individuals with neurological conditions [24], yet amalgam remains widely used in low- and middle-income settings due to its durability and cost-effectiveness.

No clinical guidelines currently recommend amalgam removal for people with MS. Small studies have reported higher mercury concentrations in urine or serum among individuals with MS compared with controls [25, 26] and anecdotal improvements following amalgam removal, accompanied by reduced lymphocyte reactivity to inorganic mercury [27]. A recent study found a significant decline in serum mercury levels after removal [28], confirming that dental fillings contribute to systemic exposure. Nevertheless, the removal procedure itself may cause a transient increase in mercury levels in biological fluids [29].

Our findings support a cautious approach to the use of amalgam in individuals at increased MS risk or with established disease, pending further evidence. Mercury is a potent neurotoxin, with known immunotoxic and nephrotoxic effects, and susceptibility may vary by genetic background [30]. Investigating gene–environment interactions may help to identify individuals most vulnerable to mercury-associated disease processes. Mercury exposure also persists in occupational and environmental contexts, including electronics manufacturing, waste handling, and artisanal gold mining, as well as through fish consumption and local emissions. These broader sources of exposure highlight the continued relevance of assessing mercury-associated health risks, particularly in susceptible groups.

Amalgam exposure is an imperfect proxy for lifetime mercury exposure that does not account for other sources. We excluded individuals with occupational mercury exposure and adjusted for fish consumption in fully adjusted models but some exposure misclassification is likely, probably biasing estimates toward the null. Nonetheless, the estimates were similar in fully adjusted and sensitivity analyses, and quantitative bias analysis indicated that relatively strong unmeasured confounding would be required to fully explain the observed associations.

Selection bias is another consideration. While nearly all cases of MS in Sweden are referred to hospital-based neurological clinics, some may not have been captured. However, given the national coverage, the proportion of unidentified cases is likely small. Control participation was lower but reported amalgam counts and smoking prevalence closely matched the national population data for similar age groups [31], suggesting minimal bias from nonresponse. Finally, although we adjusted for a broad set of covariates, some residual confounding may have remained.

In conclusion, our findings suggest that dental amalgam exposure may be associated with an increased risk of developing MS and with faster disability progression among individuals with established disease. Future studies should aim to replicate these findings by using biomarker-based measures of mercury exposure and to investigate the biological mechanisms underlying the observed associations.

## Ethics approval

The study was approved by the Regional Ethical Review Board at Karolinska Institute (reference number 2004–252/1–4 and 2013/1691–32) and was conducted in accordance with the ethical standards of the 1964 Declaration of Helsinki and its later amendments. All subjects in the study provided written informed consent before participation.

## Supplementary Material

dyag059_Supplementary_Data

## Data Availability

Anonymized data underlying this article will be shared upon reasonable request from any qualified investigator who wants to analyse questions related to the published article.
