# Supplementary material for: Dental-amalgam exposure and the risk and progression of multiple sclerosis
Source: Int J Epidemiol. 2026 Apr 24;55(3):dyag059. doi: 10.1093/ije/dyag059 (PMC13107558; doi:10.1093/ije/dyag059)
Supplement: dyag059_Supplementary_Data [file dyag059_supplementary_data.docx]

**Supplementary file - amalgam.docx**

Methods S1. Classification and categorization of covariates derived from questionnaire.

Table S1. Amalgam status by 5-year birth cohort among study participants.

Table S2. Associations between dental amalgam fillings and risk of multiple sclerosis, stratified by sex.

Table S3. Associations between number of dental amalgam fillings and risk of relapsing- versus progressive-onset multiple sclerosis.

Table S4. Baseline characteristics of participants with relapsing-onset MS born 1965-1985.

Table S5. Sociodemographic and lifestyle characteristics of controls born 1965-1985.

Table S6. Associations between dental amalgam exposure and risk of confirmed disability worsening among individuals born 1965-1985, stratified by efficacy class of first disease-modifying therapy, age at baseline, and smoking status at baseline.

Table S7. Expanded Disability Status Scale trajectories post-diagnosis by dental amalgam exposure among patients with relapsing-onset multiple sclerosis.

Table S8. Predicted mean EDSS by amalgam status.

Table S9. Sensitivity analysis: associations between number of dental amalgam fillings and risk of multiple sclerosis.

Table S10. Sensitivity analysis: associations between dental amalgam exposure and unfavorable outcomes among individuals born 1965–1985.

Figure S1. Directed acyclic graph for the total effect of dental amalgam on MS onset.

Figure S2. Directed acyclic graph for the total effect of dental amalgam on disability progression.

**Methods S1. Classification and categorization of covariates derived from questionnaire**

*Logistic regression analyses*

Ancestry: Ancestry was categorized as Nordic vs non-Nordic. A subject who was born in any of the Nordic countries, whose parents had not immigrated from outside the Nordic countries, was classified as Nordic.

Smoking: Smoking status was determined by asking participants about their current and past smoking habits. Participants who smoked at index were defined as current smokers, those who had smoked before index, but were non-smokers at index, were defined as past smokers, while those who had never smoked were categorized as never smokers.

BMI at age 20: Information was obtained regarding current body weight and height, as well as body weight at age 20. Using current height, we calculated BMI at age 20 by dividing weight in kilograms by height in meters squared. BMI was categorized as underweight, normal weight, overweight or obesity, as defined by the World Health Organization.

Past infectious mononucleosis: grouped as yes, no, or unsure.

Sun exposure: Based on three questions regarding sun exposure at diagnosis (sunbathing in Sweden, traveling to sunnier countries, and use of sunbeds), where each answer alternative was given a number ranging from 1 (the lowest exposure) to 4 (the highest exposure), we constructed an index by summing the scores (range 3-12) and dichotomized at the median into high vs low exposure.

Educational level: Educational attainment was categorized as pre-secondary (compulsory school only), secondary (completed gymnasium), or post-secondary (education beyond gymnasium, typically university). We used self-reported educational attainment, which we validated against data on formal education retrieved from Statistics Sweden.

Alcohol consumption: Alcohol consumption at diagnosis was categorized into non-drinkers, low alcohol consumption (<50 grams/week for females and <100 grams/week for males), moderate alcohol consumption (50-108 grams/week for females and 100-168 grams/week for males), and high alcohol consumption (>108 grams/week for females and >168 grams/week for males).

Fish consumption: Participants were asked about their average consumption of lean and oily fish. Responses were recorded on a 4-point scale. We constructed a frequency score for fish consumption by summing the responses, yielding a value between 2 (lowest exposure) and 8 (highest exposure).

Physical activity: Physical activity in the five years before diagnosis was categorized into low (physical activity without sweating for less than two hours per week), moderate (physical activity without sweating for at least two hours per week), moderate/high (regularly exercising for at least 30 minutes 1-2 times per week, or high (regularly exercising for at least 30 minutes at least three times per week).

*Longitudinal analyses*

Smoking: Smoking status was determined by asking participants about their current and past smoking habits. Participants who smoked at diagnosis were defined as current smokers, those who had smoked before diagnosis, but were non-smokers at diagnosis, were defined as past smokers, while those who had never smoked were categorized as never smokers.

BMI at diagnosis: Information was obtained regarding current body weight and height. Using current height, we calculated BMI by dividing weight in kilograms by height in meters squared. BMI was categorized as underweight, normal weight, overweight or obesity, as defined by the World Health Organization.

Past infectious mononucleosis: grouped as yes, no, or unsure.

Sun exposure: Based on three questions regarding sun exposure at diagnosis (sunbathing in Sweden, traveling to sunnier countries, and use of sunbeds), where each answer alternative was given a number ranging from 1 (the lowest exposure) to 4 (the highest exposure), we constructed an index by summing the scores (range 3-12) and dichotomized at the median into high vs low exposure.

Educational level: Educational attainment was categorized as pre-secondary (compulsory school only), secondary (completed gymnasium), or post-secondary (education beyond gymnasium, typically university). We used self-reported educational attainment, which we validated against data on formal education retrieved from Statistics Sweden.

Alcohol consumption: Alcohol consumption at diagnosis was categorized into non-drinkers, low alcohol consumption (<50 grams/week for females and <100 grams/week for males), moderate alcohol consumption (50-108 grams/week for females and 100-168 grams/week for males), and high alcohol consumption (>108 grams/week for females and >168 grams/week for males).

Fish consumption: Participants were asked about their average consumption of lean and oily fish. Responses were recorded on a 4-point scale. We constructed a frequency score for fish consumption by summing the responses, yielding a value between 2 (lowest exposure) and 8 (highest exposure).

Physical activity: Physical activity at diagnosis was categorized into low (physical activity without sweating for less than two hours per week), moderate (physical activity without sweating for at least two hours per week), moderate/high (regularly exercising for at least 30 minutes 1-2 times per week, or high (regularly exercising for at least 30 minutes at least three times per week).

**Table S1. Amalgam status by 5-year birth cohort among study participants.**

| **Birthyear** | **Number of amalgam fillings** | | |
| --- | --- | --- | --- |
|  | 0 | 1-5 | 6 or more |
| <1960 | 9 (3.4) | 29 (10.8) | 231 (85.9) |
| 1960-1964 | 6 (2.7) | 33 (14.9) | 182 (82.4) |
| 1965-1969 | 17 (6.2) | 85 (30.9) | 173 (62.9) |
| 1970-1974 | 50 (16.1) | 134 (43.1) | 127 (40.8) |
| 1975-1979 | 115 (37.6) | 145 (47.4) | 46 (15.0) |
| 1980-1984 | 204 (80.0) | 40 (15.7) | 11 (4.3) |
| 1985-1990 | 167 (93.8) | 10 (5.6) | 1 (0.6) |
| 1990- | 52 (100) | 0 | 0 |

Participants born between 1965-1985 (grey-shaded rows) were included in longitudinal analyses

**Table S2.** Associations between dental amalgam fillings and risk of multiple sclerosis, stratified by sex.

| **Women** | | | | |
| --- | --- | --- | --- | --- |
| No. of fillings | Cases/controls | OR (95% CI)^1^ | OR (95% CI)^2^ | p |
| 0 | 355/921 | 1.0 (reference) | 1.0 (reference) |  |
| 1-5 | 307/640 | 1.17 (1.00-1.38) | 1.19 (1.01-1.40) |  |
| 6-10 | 340/716 | 1.22 (1.02-1.45) | 1.29 (1.08-1.54) |  |
| 11-14 | 166/340 | 1.31 (1.05-1.63) | 1.35 (1.08-1.68) |  |
| 15- | 86/156 | 1.63 (1.23-2.16) | 1.76 (1.32-2.34) | 1.14 (1.07-1.21) |
| **Men** | | | | |
| No. of fillings | Cases/controls | OR (95% CI)^1^ | OR (95% CI)^2^ | p |
| 0 | 137/354 | 1.0 (reference) | 1.0 (reference) |  |
| 1-5 | 114/261 | 1.19 (0.92-1.55) | 1.21 (0.93-1.58) |  |
| 6-10 | 135/268 | 1.29 (0.98-1.71) | 1.33 (1.00-1.78) |  |
| 11-14 | 54/109 | 1.31 (0.91-1.88) | 1.34 (0.92-1.94) |  |
| 15- | 46/62 | 1.82 (1.19-2.80) | 1.79 (1.16-2.76) | 1.15 (1.04-1.26) |

OR=odds ratio; CI=confidence intervals; ^1^adjusted for age at disease onset, residential area (according to study design), and ancestry; ^2^adjusted for age at disease onset, residential area (according to study design), ancestry, smoking status at disease onset, past infectious mononucleosis, body mass index at age 20, and sun exposure habits.

**Table S3.** Associations between number of dental amalgam fillings and risk of relapsing- versus progressive-onset multiple sclerosis

| **Relapsing-remitting MS** | | | | |
| --- | --- | --- | --- | --- |
| Nb of fillings | ca/co* | OR (95% CI)^1^ | OR (95% CI)^2^ | OR for trend (95% CI) |
| 0 | 694/1694 | 1.0 (reference) | 1.0 (reference) |  |
| 1-5 | 517/1130 | 1.12 (0.96-1.36) | 1.22 (1.06-1.41) |  |
| 6-10 | 523/1178 | 1.25 (1.07-1.46) | 1.29 (1.10-1.50) |  |
| 11-14 | 242/539 | 1.31 (1.08-1.60) | 1.32 (1.09-1.61) |  |
| 15- | 134/241 | 1.64 (1.28-2.10) | 1.70 (1.32-2.08) | 1.18 (1.07-1.29) |
| **Progressive-onset MS** | | | | |
| Nb of fillings | ca/co* | OR (95% CI)^1^ | OR (95% CI)^2^ | OR for trend (95% CI) |
| 0 | 10/1694 | 1.0 (reference) | 1.0 (reference) |  |
| 1-5 | 19/1130 | 1.14 (0.57-2.30) | 1.11 (0.56-2.20) |  |
| 6-10 | 34/1178 | 1.29 (0.66-2.50) | 1.26 (0.64-2.45) |  |
| 11-14 | 23/539 | 1.58 (0.76-3.26) | 1.54 (0.74-3.12) |  |
| 15- | 16/241 | 2.26 (1.03-4.97) | 2.18 (1.00-4.23) | 1.30 (1.03-1.63) |

OR=odds ratio; CI=confidence interval; ^1^adjusted for age at disease onset, sex, residential area (according to study design), and ancestry; ^2^adjusted for age at disease onset, sex, residential area (according to study design), ancestry, smoking status at disease onset, past infectious mononucleosis, body mass index at age 20, and sun exposure habits.

**Table S4**. Baseline characteristics of participants with relapsing-onset MS born 1965-1985.

|  | Total | Amalgam exposure | | |
| --- | --- | --- | --- | --- |
|  |  | 0 | 1-5 | 6 or more |
| N | 1191 | 408 | 409 | 374 |
| Age at index (SD) | 31.5 (6.4) | 29.3 (5.4) | 32.8 (5.9) | 33.7 (6.2) |
| Age at diagnosis (SD) | 34.0 (6.0) | 31.2 (5.1) | 34.9 (5.3) | 35.8 (5.0) |
| Women (n, %) | 875 (73.5) | 293 (71.8) | 303 (74.1) | 279 (74.6) |
| Nordic (n, %) | 957 (80.4) | 314 (77.0) | 331 (80.9) | 312 (83.4) |
| Post-secondary education, n (%) | 578 (48.5) | 214 (52.5) | 201 (49.1) | 163 (43.6) |
| Mean EDSS (SD) | 1.6 (1.3) | 1.5 (1.3) | 1.6 (1.3) | 1.7 (1.3) |
| Treatment regimen: None, n (%) Platform DMT, n (%) Escalation therapy, n (%)  High-efficacy DMT, n (%) | 54 (4.5) 348 (29.2) 631 (53.0) 158 (13.3) | 26 (6.4) 96 (23.5) 225 (55.1) 61 (15.0) | 7 (1.7) 123 (30.1) 225 (55.0) 54 (13.2) | 21 (5.6) 127 (34.0) 187 (50.0) 39 (10.4) |
| Proportion of follow-up on DMT (SD) | 0.9 (0.2) | 0.9 (0.2) | 0.9 (0.2) | 0.9 (0.3) |
| Past IM, n (%) No past IM, n (%) Unsure, n (%) | 264 (22.2) 822 (69.1) 103 (8.7) | 102 (25.1) 267 (65.8) 37 (9.1) | 87 (21.3) 296 (72.4) 26 (6.4) | 75 (20.1) 259 (69.3) 40 (10.7) |
| Never smoking, n (%) Current smoking, n (%) Past smoking, n (%) | 610 (51.2) 331 (27.8) 250 (21.0) | 212 (52.0) 106 (26.0) 90 (22.1) | 218 (53.3) 113 (27.6) 78 (19.1) | 180 (48.1) 112 (30.0) 82 (21.9) |
| Alcohol use, n (%) Gram alcohol/week (SD) | 824 (69.2) 46.5 (64.3) | 269 (65.9) 41.8 (57.4) | 286 (69.9) 44.3 (64.1) | 269 (71.9) 28.1 (35.0) |
| Fish consumption score (SD) | 3.8 (1.1) | 3.8 (1.1) | 3.8 (0.8) | 3.7 (1.1) |
| Underweight, n (%) Normal weight, n (%) Overweight, n (%) Obesity, n (%) | 41 (3.4) 694 (58.3) 296 (24.9) 160 (13.4) | 17 (4.2) 245 (60.1) 91 (22.3) 55 (13.5) | 12 (2.9) 246 (60.2) 99 (24.2) 52 (12.7) | 12 (3.2) 203 (54.3) 106 (28.3) 53 (14.2) |
| Sun exposure score (SD) | 6.3 (1.9) | 6.4 (1.8) | 6.4 (1.9) | 6.3 (1.9) |
| Regular physical activity, n (%) | 441 (37.0) | 163 (40.0) | 160 (39.1) | 118 (31.6) |

SD=standard deviation; EDSS=expanded disability status scale; DMT=disease-modifying therapy, IM=infectious mononucleosis.

**Table S5**. Sociodemographic and lifestyle characteristics of controls born 1965-1985.

|  | Total | Amalgam exposure | | |
| --- | --- | --- | --- | --- |
|  |  | 0 | 1-5 | 6 or more |
| N | 2912 | 1194 | 884 | 834 |
| Age at index (SD) | 31.3 (6.6) | 28.2 (5.7) | 32.1 (6.3) | 34.8 (6.1) |
| Women (n, %) | 2121 (72.8) | 854 (71.5) | 644 (72.9) | 623 (74.7) |
| Nordic (n, %) | 2238 (76.9) | 855 (71.6) | 693 (78.4) | 690 (82.7) |
| Post-secondary education, n (%) | 1436 (50.7) | 586 (49.1) | 468 (53.0) | 382 (45.8) |
| Past IM, n (%) No past IM, n (%) Unsure, n (%) | 319 (10.9) 2300 (79.0) 94 (10.1) | 140 (11.7) 942 (78.9) 112 (9.4) | 89 (10.1) 708 (80.1) 87 (9.8) | 90 (10.8) 650 (77.9) 94 (11.3) |
| Never smoking, n (%) Current smoking, n (%) Past smoking, n (%) | 1740 (59.8) 667 (22.9) 504 (17.3) | 706 (59.1) 298 (25.0) 190 (15.9) | 552 (62.5) 193 (21.9) 138 (15.6) | 482 (57.8) 176 (21.1) 176 (21.1) |
| Alcohol use, n (%) Gram alcohol/week (SD) | 1999 (68.9) 50.2 (75.2) | 797 (66.8) 51.9 (81.6) | 601 (68.0) 45.6 (64.1) | 601 (72.1) 53.2 (77.3) |
| Fish consumption score (SD) | 3.8 (1.1) | 3.8 (1.1) | 3.9 (1.1) | 3.9 (1.1) |
| Underweight, n (%) Normal weight, n (%) Overweight, n (%) Obesity, n (%) | 20 (3.4) 1702 (58.5) 279 (28.3) 97 (9.9) | 48 (4.0) 742 (62.1) 294 (24.6) 110 (9.2) | 32 (3.6) 522 (59.0) 250 (28.3) 80 (9.1) | 20 (2.4) 438 (52.5) 279 (33.5) 97 (11.6) |
| Sun exposure score (SD) | 6.7 (2.0) | 6.8 (2.0) | 6.5 (1.9) | 6.7 (2.0) |
| Regular physical activity, n (%) | 1615 (55.5) | 683 (57.2) | 478 (54.1) | 454 (54.4) |

SD=standard deviation; IM=infectious mononucleosis.

**Table S6**. Associations between dental amalgam exposure and risk of confirmed disability worsening among individuals born 1965-1985, stratified by efficacy class of first disease-modifying therapy, age at baseline, and smoking status at baseline.

|  | Only low-efficacy treatment | | High-efficacy treatment | | |
| --- | --- | --- | --- | --- | --- |
| Amalgam | HR (95% CI)^1^ | HR (95% CI)^2-3^ | HR (95% CI)^1^ | HR (95% CI)^2-3^ | |
| 0 | 1.0 (reference) | 1.0 (reference) | 1.0 (reference) | 1.0 (reference) | |
| 1-5 | 1.27 (0.84-1.94) | 1.19 (0.76-1.85) | 1.13 (0.90-1.41) | 1.10 (0.86-1.39) | |
| 6 or more | 1.64 (1.09-2.45) | 1.63 (1.02-2.60) | 1.33 (1.06-1.68) | 1.28 (1.00-1.68) | |
| Age at diagnosis <40 | | | Age at diagnosis >40 | | |
| Amalgam | HR (95% CI)^1^ | HR (95% CI)^2-4^ | HR (95% CI)^1^ | | HR (95% CI)^2-4^ |
| 0 | 1.0 (reference) | 1.0 (reference) | 1.0 (reference) | | 1.0 (reference) |
| 1-5 | 1.09 (0.89-1.34) | 1.09 (0.88-1.36) | 2.09 (0.89-4.89) | | 2.27 (0.95-5.44) |
| 6 or more | 1.30 (1.04-1.61) | 1.30 (1.01-1.67) | 2.22 (0.97-5.10) | | 2.52 (1.08-5.91) |
| Current smokers | | | Non-smokers | | |
| Amalgam | HR (95% CI)^1^ | HR (95% CI)^2, 4^ | HR (95% CI)^1^ | | HR (95% CI)^2, 4^ |
| 0 | 1.0 (reference) | 1.0 (reference) | 1.0 (reference) | | 1.0 (reference) |
| 1-5 | 1.50 (0.98-2.28) | 1.50 (0.93-2.43) | 1.05 (0.84-1.31) | | 1.05 (0.83-1.32) |
| 6 or more | 1.58 (1.03-2.42) | 1.89 (1.09-3.27) | 1.30 (1.04-1.62) | | 1.28 (1.00-1.66) |

CDW=confirmed disability worsening; DMT=disease-modifying treatment; HR=hazard ratio; CI=confidence interval; ^1^adjusted for baseline age and sex; ^2^adjusted for baseline age, sex, disease duration, baseline EDSS, past infectious mononucleosis, body mass index at diagnosis, sun exposure habits, and the proportion of follow-up on DMT. ^3^adjusted for baseline smoking status, ^4^adjusted for efficacy class of initial DMT.

**Table S7.** Expanded Disability Status Scale trajectories post-diagnosis by dental amalgam exposure among patients with relapsing-onset multiple sclerosis

| **Parameters** | **β coefficient (95% CI)** | ***P-value*** |
| --- | --- | --- |
| Intercept | 1.26 (0.75, 1.77) | <.0001 |
| Time | -0.07 (-0.12, -0.03) | 0.001 |
| **Number of amalgam fillings** | | |
| 0 | Reference |  |
| 1-5 | 0.04 (-0.15, 0.22) | 0.697 |
| 6 or more | 0.16 (-0.05, 0.37) | 0.131 |
| **Number of amalgam fillings* Time** | | |
| 0 | Reference |  |
| 1-5 | 0.01 (-0.01, 0.03) | 0.269 |
| 6 or more | 0.02 (0.001, 0.04) | 0.044 |

Models were adjusted for baseline age, sex, disease duration, baseline EDSS, DMT exposure, baseline smoking status, past infectious mononucleosis, body mass index at diagnosis, sun exposure habits.

**Table S8.** Predicted mean EDSS by amalgam status

| Amalgam fillings | Predicted mean EDSS at 5 years (95% CI) |
| --- | --- |
| 0 | 1.64 (1.47-1.82) |
| 1-5 | 1.73 (1.58-1.88) |
| 6 or more | 1.91 (1.75-2.07) |
| Amalgam fillings | Predicted mean EDSS at 10 years (95% CI) |
| 0 | 1.70 (1.49-1.91) |
| 1-5 | 1.84 (1.66-2.01) |
| 6 or more | 2.07 (1.88-2.26) |
| Amalgam fillings | Predicted mean EDSS at 15 years (95% CI) |
| 0 | 1.76 (1.50-2.02) |
| 1-5 | 1.94 (1.72-2.17) |
| 6 or more | 2.23 (2.00-2.46) |

Predictions derived from the adjusted linear mixed-effects model; EDSS=expanded disability status scale; CI=confidence interval.

**Table S9.** Sensitivity analysis: associations between number of dental amalgam fillings and risk of multiple sclerosis.

| Nb of fillings | Cases/controls | OR (95% CI)^1^ | OR (95% CI)^2^ | OR for trend (95% CI) |
| --- | --- | --- | --- | --- |
| 0 | 742/1694 | 1.0 (reference) | 1.0 (reference) | 1.14 (1.08-1.20) |
| 1-5 | 560/1130 | 1.18 (1.02-1.35) | 1.24 (1.07-1.44) |  |
| 6-10 | 597/1178 | 1.24 (1.07-1.43) | 1.34 (1.15-1.57) |  |
| 11-14 | 283/539 | 1.31 (1.08-1.58) | 1.36 (1.11-1.67) |  |
| 15- | 162/241 | 1.68 (1.33-2.12) | 1.75 (1.36-2.26) |  |

OR=odds ratio; CI=confidence interval; ^1^adjusted for age at disease onset, sex, residential area (according to study design), and ancestry; ^2^adjusted for age at disease onset, sex, residential area (according to study design), ancestry, smoking status at disease onset, past infectious mononucleosis, body mass index at age 20, sun exposure habits, educational attainment, alcohol consumption, fish consumption, and physical activity.

**Table S10**. Sensitivity analysis: associations between dental amalgam exposure and unfavorable outcomes among individuals born 1965-1985.

| CDW | | | | | |
| --- | --- | --- | --- | --- | --- |
| Amalgam | N | Years (SD) | Outcome (%) | HR (95% CI)^1^ | HR (95% CI)^2^ |
| 0 | 455 | 7.3 (4.7) | 215 (47) | 1.0 (reference) | 1.0 (reference) |
| 1-5 | 410 | 7.3 (4.9) | 224 (55) | 1.13 (0.93-1.37) | 1.12 (0.91-1.38) |
| 6 or more | 374 | 6.7 (4.8) | 223 (60) | 1.35 (1.11-1.64) | 1.34 (1.06-1.68) |
| EDSS 3 | | | | | |
| Amalgam | N | Years (SD) | Outcome (%) | HR (95% CI)^1^ | HR (95% CI)^2^ |
| 0 | 375 | 8.8 (4.6) | 106 (28) | 1.0 (reference) | 1.0 (reference) |
| 1-5 | 345 | 8.5 (5.0) | 128 (37) | 1.34 (1.03-1.76) | 1.39 (1.12-2.00) |
| 6 or more | 295 | 8.8 (5.0) | 116 (39) | 1.37 (1.04-1.81) | 1.48 (1.07-2.05) |
| EDSS 4 | | | | | |
| Amalgam | N | Years (SD) | Outcome (%) | HR (95% CI)^1^ | HR (95% CI)^2^ |
| 0 | 375 | 10.4 (4.0) | 36 (10) | 1.0 (reference) | 1.0 (reference) |
| 1-5 | 345 | 10.4 (4.5) | 56 (16) | 1.61 (1.04-2.48) | 1.72 (1.08-2.74) |
| 6 or more | 295 | 10.6 (4.6) | 51 (17) | 1.60 (1.02-2.51) | 1.75 (1.03-2.96) |

HR=hazard ratio; CI=confidence interval; CDW=confirmed disability worsening; EDSS=expanded disability status scale; ^1^adjusted for baseline age and sex; ^2^adjusted for baseline age, sex, disease duration, baseline EDSS, DMT exposure, baseline smoking status, past infectious mononucleosis, body mass index at diagnosis, sun exposure habits, educational attainment, alcohol use, fish consumption, and physical activity.

Figure S1. Directed acyclic graph for the total effect of dental amalgam on MS onset


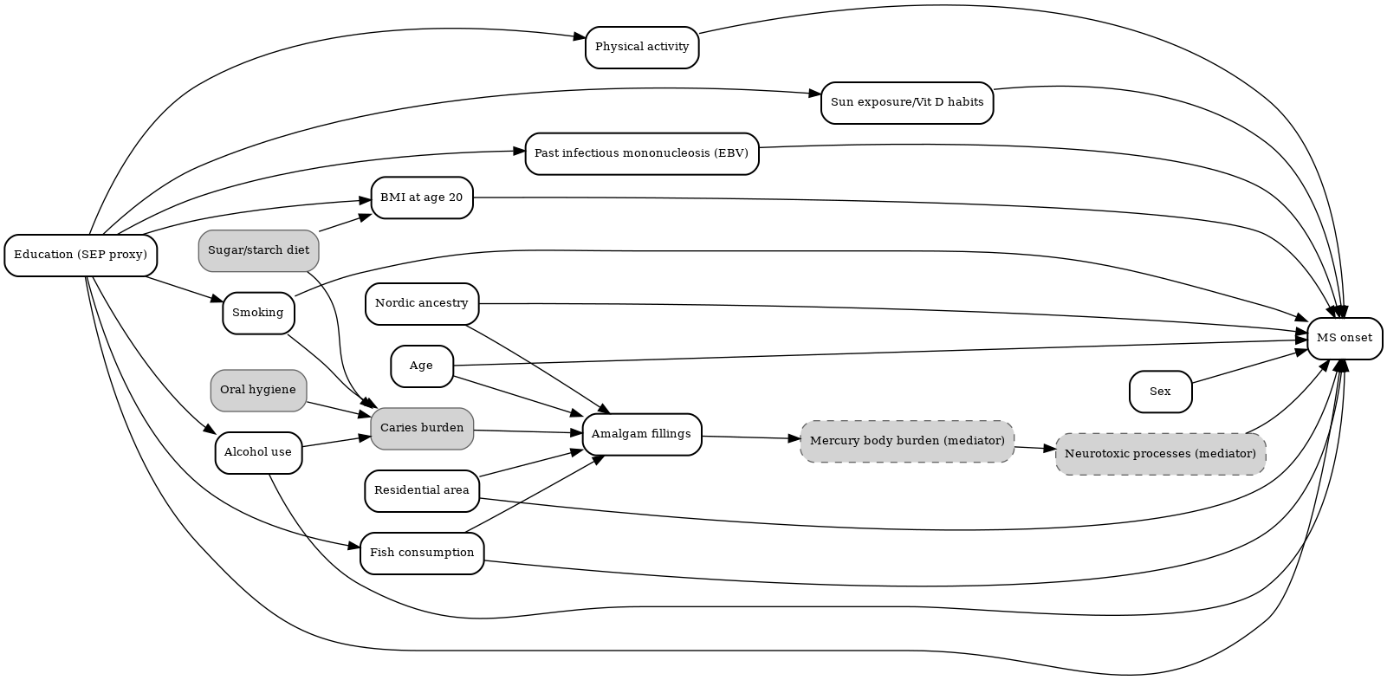


Nodes shown in white with bold borders were measured in EIMS; light-gray nodes denote unmeasured constructs; dashed borders indicate mediators that were not adjusted for. Arrows represent assumed causal directions based on prior knowledge. The estimand is the total effect of amalgam fillings on MS onset. The minimal sufficient adjustment set comprised age, sex, residential area (matching factors), ancestry, smoking, bod mass index at age 20, sun exposure habits, and prior infectious mononucleosis. Education (proxy for socioeconomic position), fish and alcohol consumption, and physical activity were evaluated in fully adjusted models. Upstream determinants (diet, oral hygiene, caries) are shown to justify confounding control but were not directly adjusted where doing so could induce bias via conditioning on colliders. Mediators (mercury body burden, neurotoxic processes) were not adjusted for.

Figure S2. Directed acyclic graph for the total effect of dental amalgam on disability progression


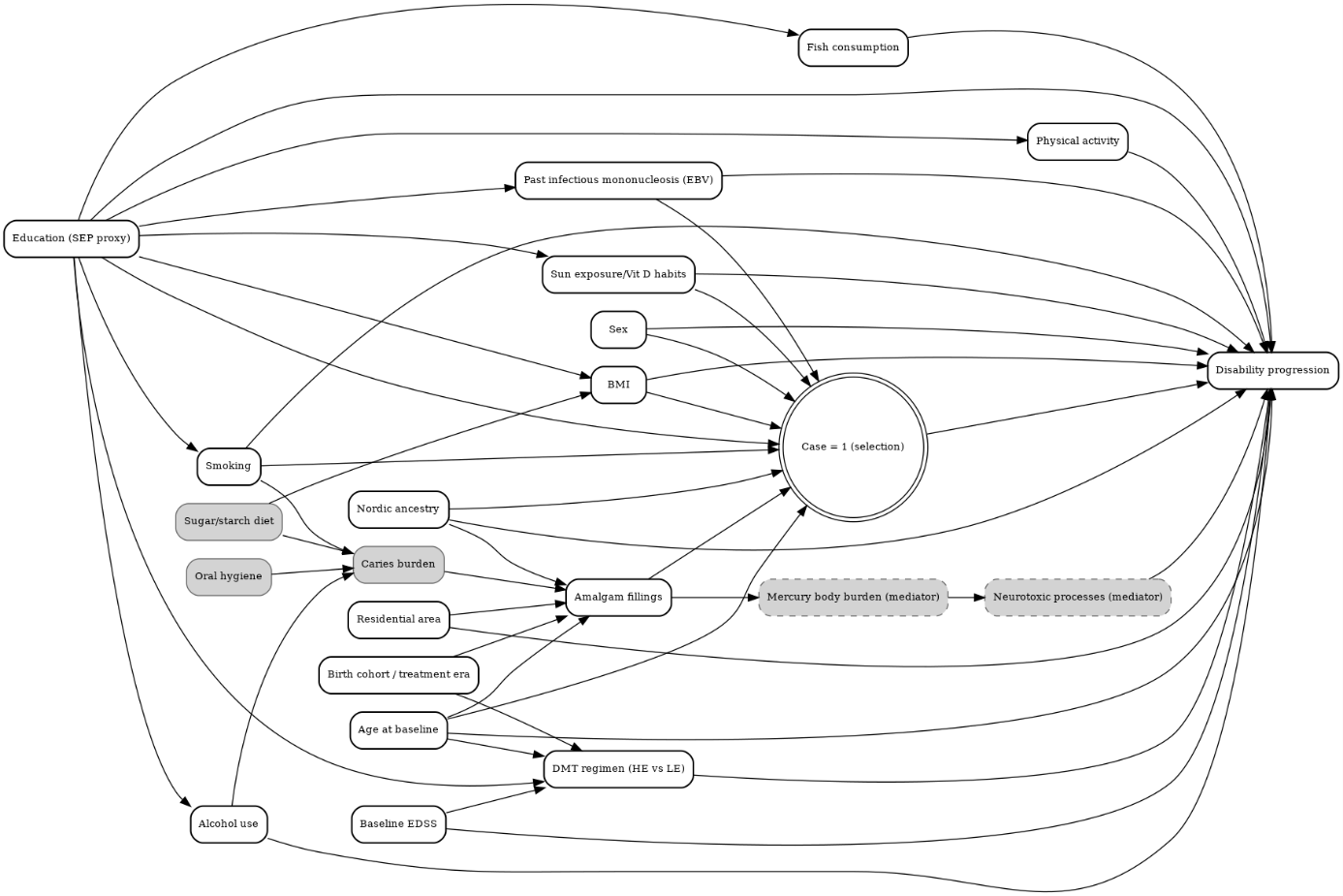


Nodes in white were measured; light-gray nodes are unmeasured; dashed borders indicate mediators not adjusted for. The double-circle “Case=1” denotes conditioning on case status. The estimand is the total effect of amalgam on disability progression among cases. Confounding control included age at baseline, sex, ancestry, disease duration, baseline EDSS, disease-modifying therapy, smoking, sun exposure habits and prior infectious mononucleosis. Birth cohort/treatment era is shown since it affects both amalgam prevalence and access to high-efficacy DMT, addressed by restricting to birth cohorts 1965-1985. Mediators (mercury body burden, neurotoxic processes) were not adjusted for. EDSS=expanded disability status scale; DMT=disease modifying treatment.
